# On the Linguistic and Cognitive Factors that Predict Reading Abilities Among Arabic-Speaking University Students

**DOI:** 10.1007/s10936-026-10238-9

**Published:** 2026-04-10

**Authors:** Bahaa Madi Tarabya, Asaid Khateb

**Affiliations:** 1 The Unit for the Study of Arabic Language, Edmond J. Safra Brain Research Center for the Study of Learning Disabilities, University of Haifa, 199 Abba Khoushy Ave., Mount Carmel, 3498838 Haifa, Israel; 2 Dept of Learning Disabilities, Faculty of Education, University of Haifa, Haifa, Israel

**Keywords:** Reading predictors, Reading accuracy, Reading rate, Reading comprehension, Adult readers

## Abstract

While research on the predictors of reading and reading comprehension is extensive in child and in some adult populations, particularly in alphabetic languages, few studies have examined such factors among Arabic-speaking adults. This study examined the extent to which the same linguistic and cognitive predictors contribute to reading accuracy, rate and comprehension among university students. A total of 120 young adults (aged 18–32) completed a battery of tests to examine their reading skills, phonological awareness (PA), rapid automatized naming (RAN), morphological knowledge, executive functions and verbal long-term memory. Stepwise regression analyses were conducted on reading abilities. We hypothesized that PA, RAN, morphological awareness, and working memory will significantly predict reading accuracy and fluency, and to some extent, reading comprehension. Our results indicated that PA emerged as the strongest predictor of reading accuracy, while RAN most significantly predicted reading speed. Reading comprehension, however, was best predicted by a combination of phonological and morphological abilities, along with inhibitory control and long-term memory. These findings fill a gap in literature and shed light on the linguistic and cognitive mechanisms underlying adult reading skills in Arabic, a language with a complex orthographic system and diglossic situation. The results of this study are discussed in the light of previous reading literature. We propose that additional research, including other linguistic, cognitive and psycho-social factors, will be needed to gain a deeper understanding of the interplay between factors that influence reading in a language with unique linguistic features.

## Introduction

Reading is often defined as “a complex cognitive process of decoding printed symbols to derive meaning” (Rayner et al., 2001). This process involves not only recognizing written words but also efficiently integrating background knowledge, linguistic skills, and cognitive abilities in the construction of meaning from written texts (Landerl et al., 2019; Perfetti & Stafura, 2014; Ziegler et al., 2010). Reading ultimately aims to enable comprehension, which allows the reader to interpret, analyze, and understand the content and context of written texts. For a very long time, reading research has mainly been conducted on English and other alphabetic European languages. However, because English represents an extreme case of spelling-sound ambiguity, and thus could be considered an outlier language, Share (2008) had emphasized the necessity to investigate literacy processes in other languages while taking into account the unique features of their writing and linguistic systems in order to “*extricate reading science from entrenched Anglocentricism*”(Share, 2008, 2014, 2020, 2021). In contrast with the central processing approach (Coltheart, 2005; Patterson et al., 1996; Seidenberg & McClelland, 1989), Share’s view aligns with the script-dependent approach assuming that the same cognitive factors might contribute differently to reading due to the specific characteristics of the writing systems (Bick et al., 2011; Geva & Siegel, 2000) and to their orthographic transparency (e.g., Lundberg et al., 1988; Pennington et al., 1990; Share, 2008). This latter view might be particularly relevant for the Semitic Arabic language which is characterized by several particular features (see some details hereafter Saiegh-Haddad & Henkin-Roitfarb, 2014).

The study of reading abilities\disabilities has historically concentrated on reading accuracy in which phonological processing is considered as a very important factor that significantly determine differences between many dyslexic and normal readers (Jorm & Share, 1983; Liberman et al., 1989; Snowling et al., 2020; Stanovich, 2000; Wagner & Torgesen, 1987) and thus is at the basis of reading capacity (Lyon et al., 2003; Shaywitz & Shaywitz, 2020; Snowling et al., 2000). The quality of phonological processing is thought to be very crucial for the development of phonemic awareness (PA) which is a major predictor of reading acquisition in various languages (Bishop & Adams, 1990; Mahfoudhi et al., 2011; Saiegh-Haddad & Geva, 2008; Snowling et al., 2000; Stanovich, 1988; Ziegler & Goswami, 2005; Ziegler et al., 2010). PA defines the individual’s knowledge that words are made up of sound segments and small units of speech sounds, and that spoken words/speech can be segmented into phonemic units (Foorman et al., 1997; Hoover & Gough, 1990; Nicholson, 1997). The critical role played by PA during the very early phases of *learning to read* is because the child needs to internalize the relations between the phonemes and the graphemes (i.e., the orthographic symbols) and thus acquire the so-called “*grapho-phonemic conversion (GPC) rules*”. The conversion of orthographic into phonetic code (Tunmer & Rohl, 1991; Zifcak, 1981), and the knowledge of how these can be combined to construct words (Bentin & Leshem, 1993) by systematically mapping and blending the phonological elements within words determine the child’s word decoding abilities (Perfetti, 2007; Perfetti et al., 1987). Authors had suggested that the role of PA in learning to read diminish with the development of the alphabetic code, but remains more important in deep than in transparent orthographies (Shatil & Share, 2003).

### On the Major Predictors of Reading Development

A considerable shift has occurred during the last two decades in the manner authors look at reading determinants. Despite the consensus that impairment in PA constitutes the core deficit in many reading difficulties, evidence had shown the phonological deficits are not sufficient to explain all reading disabilities (Nation & Snowling, 2004; Share, 1995). Nowadays, it is commonly recognized that reading difficulties might include either inaccurate or dysfluent reading or both, highlighting the fact that reading speed is an important characteristic of reading abilities/disabilities (Leinonen et al., 2001; Lovett, 1984a, 1984b, 1987; Shany & Share, 2011). In this regard, it had been argued that rapid automatized naming (RAN, Denckla & Rudel, 1976), seen as an index of the phonological abilities (Wagner et al., 1994) or as a domain-specific index of speed of information processing (Breznitz, 2006), constitutes an additional independent factor predicting reading, consequently as an additional risk factor for dyslexia (see also Pennington et al., 2001; Wolf & Bowers, 1999; Wolf et al., 2002). Research indeed showed that PA and RAN do not tap a single and common phonological construct (Wolf & Bowers, 2000) and performance in these tasks generally show weak correlations (Asadi et al., 2017a, 2017b; Bowers & Wolff, 1993; Swanson et al., 2003). Studies on reading in English and other orthographies have found that RAN was more strongly related to fluency than to accuracy, whereas PA was more strongly related to accuracy than to fluency (Moll et al., 2014; see for a review Norton & Wolf, 2012). Hence, current views on reading acquisition suggest that the linguistic and cognitive skills that contribute to reading accuracy may differ from those participating to reading fluency and reading comprehension (Shany & Breznitz, 2011; Verhoeven et al., 2011).

The literature dealing with reading development indicates that, in addition to PA and RAN (Caravolas et al., 2005, 2012; Defior, 2004; Goswami, 1999; Landerl et al., 2019; López-Escribano et al., 2018; McWeeny et al., 2022; Pfost, 2015; Zugarramurdi et al., 2022), several other linguistic and cognitive factors play important roles in reading acquisition both in alphabetical and non-alphabetical languages. Among the other major predictors of reading, it is worth mentioning the following:

*Orthographic knowledge* (Apel & Lawrence, 2011), defined as word-specific and general orthographic knowledge, had been shown to contribute to reading in many different languages (see foe English Conrad et al., 2013; see also, Zhao et al., 2017), Portuguese (Querido et al., 2021), German (Zarić et al., 2021), Chinese (Lin et al., 2019; Siok & Fletcher, 2001), Hebrew (Schiff, 2012) and Arabic (Asadi et al., 2017a, 2017b).

*Morphological knowledge or morphological awareness (hereafter MA)* refers to the ability to consciously recognize and understand the units of meaning within words and the relationship between base words and their inflected and derived forms. Morphological knowledge has been shown to predict reading in the deep English orthography (Apel & Lawrence, 2011; Deacon & Kirby, 2004; Elbro & Arnbak, 1996) but also in various other languages as for example, Portuguese (de Freitas et al., 2018), French (Quémart et al., 2012), Italian (Casalis et al., 2015; Dumay et al., 2002), Hebrew (Cohen-Mimran et al., 2023) and Arabic (Asadi et al., 2017a, 2017b; Saiegh‐Haddad & Taha, 2017; Schiff & Saiegh-Haddad, 2018).

*Vocabulary knowledge* has also been consistently shown to be a strong predictor of reading ability in English (in particular for reading irregular words) and others languages (Manolitsis et al., 2017; Ouellette, 2006; Ricketts et al., 2007; Wise et al., 2007) including Arabic (Asadi & Khateb, 2017; Asadi et al., 2017a, 2017b) and Hebrew (Shahar-Yames & Prior, 2017).

*Executive functions (EFs)*, defined as a range of cognitive processes that determine the individual’s ability to regulate mental functions and engage in goal directed behavior (Denckla, 1994), are grouped in three main domains including: working memory updating, shifting, and inhibitory control (Miyake et al., 2000). Short term and working memory more particularly (Baddeley, 2000) had been shown to be associated with reading and reading comprehension in various language (see meta analysis, Carretti et al., 2009; Yeniad et al., 2013) including English (Altemeier et al., 2008; Blair & Razza, 2007; Jacobson et al., 2017; Miller et al., 2014; Welsh et al., 2010), Chinese (Chung & McBride-Chang, 2011), Dutch (Segers et al., 2016), Portuguese (Engel de Abreu et al., 2014), Hebrew (Bental & Tirosh, 2007) and Arabic (Asadi & Khateb, 2017).

### On the Major Predictors of Reading Comprehension

The development of reading comprehension (RC), as the ultimate goal of reading acquisition, has also attracted many researchers in various languages and led to the proposition of different theoretical models (Aaron et al., 2008; Gough & Tunmer, 1986; Joshi & Aaron, 2000). The "*Simple View of Reading*" (SVR) model, one of the most prevalent theories on RC, first conceptualized for English by Gough and Tunmer (1986) and expanded by Hoover and Gough (1990), proposes that RC can be explained by decoding and listening comprehension abilities. Thus, the SVR suggests that readers who have not yet fully developed the ability to decode words quickly and accurately, or to extract meaning from discourse, will encounter difficulties in comprehending written text. Various studies across different languages have tested the validity of this model (Asadi et al., 2017a, 2017b; Braze et al., 2016; Cervetti et al., 2020; Hoover, 2023; Johnston & Kirby, 2006; Joshi et al., 2015; Language & Consortium, 2015; Language et al., 2018; Lonigan et al., 2018; Tilstra et al., 2009) and found that the amount of explained variance could differ between language depending on their orthographic depth. In a study by (Joshi et al., 2012) the validity of the SVR was compared in Spanish (as a transparent orthography), Chinese and English. The observation that the model explained more variance in reading comprehension in Spanish (~ 60%) than in English (~ 50%) and in Chinese (between 25 and 42%). The authors explained these findings by noting that different factors accounted for variance differently across languages; for example, decoding explained less variance in the relatively transparent Spanish orthography than in the deeper English orthography. Joshi et al. (2015) tested the validity of the SVR in Hebrew (a Semitic Language) amongst grades 2 to 10 readers. They found that decoding and listening comprehension could explain up to 70% of the variance in RC. Joshi and colleagues (Aaron et al., 2008; Joshi et al., 2008, 2012) expanded the SVR into the *Componential Model of Reading* (CMR) arguing that, although both decoding and listening comprehension were necessary for reading comprehension, neither was sufficient alone. Similar to the RAND approach (Sweet, 2005), besides linguistic and cognitive factors, the CMR includes psychological, and ecological components such as motivation, home environment, and parental involvement. A validation investigation of the extended SVR has been tested among first to sixth grade Arabic-speaking children (Asadi et al., 2017a, 2017b).

### An Overview of Some of the Specific Features of Arabic Language

Arabic is characterized by several unique features including a diglossic situation, a visually complex orthographic system and a rich morphological system (Eberhard et al., 2020; Elbeheri, 2023; Khateb & Ibrahim, 2022; Saiegh-Haddad, 2020). These characteristics are thought to represent unique challenges to young and adult readers. The diglossic situation (Ferguson, 1959) refers to the existence of a socio-linguistic situation in which two varieties of the same language are used for different purposes: Spoken Arabic (SA), used in everyday conversation and informal communication purposes and literary Arabic (LA) which is formally acquired at schools through formal education for reading and writing and used more for formal purposes (Saiegh-Haddad, 2020; Saiegh-Haddad & Joshi, 2014; Saiegh-Haddad & Schiff, 2016; Saiegh-Haddad & Spolsky, 2014). Since LA differs from SA in almost all its linguistic aspects including the grammatical, syntactic, morphological, phonological and lexical aspects, diglossia is considered a major challenge for literacy acquisition in Arabic (Al Ghanem & Kearns, 2015; Mahfoudhi et al., 2011; Saiegh-Haddad, 2005, 2013; Saiegh-Haddad & Joshi, 2014). This challenge is often attributed to difficulties in building stable phonological representations for LA words, requiring children to acquire both new linguistic-auditory and orthographic-visual systems during early schooling (Ibrahim et al., 2002; Khateb & Ibrahim, 2022; Saiegh-Haddad, 2004).

Arabic is characterized by an orthographic system that makes reading and writing acquisition particularly challenging compared to many other languages. It is an “*abjad*” consonantal system written from right to left (Daniels & Share, 2018; Saiegh-Haddad & Joshi, 2014) consisting of 28 consonant letters, of which three serve also as long vowels. Short vowels are indicated by diacritics added above and below letters and provide critical phonological and morpho-syntactic information (Saiegh-Haddad & Schiff, 2016). The presence or absence of short vowels determines orthographic depth, such that (short) vowelized Arabic is relatively transparent, whereas unvowelized Arabic is deep and highly homographic (Abu-Rabia, 2001; Frost et al., 2005; Saiegh-Haddad & Schiff, 2016). Orthographic complexity is further increased by visually similar graphemes distinguished only by dots, as well as by letter-shape variations depending on within-word connectivity (Asadi et al., 2017a, 2017b; Khateb et al., 2013, 2014). These combined features contribute to difficulties in word recognition and spelling, particularly in early stages of literacy development (Khateb et al., 2014; Yassin et al., 2020).

Finally, the complexity of written Arabic is further augmented by the rich and dense derivational and inflectional morphological system. Morphological complexity in Arabic stems from the fact that a single Arabic word can include several morphemes, such that one single Arabic word might correspond to one complete sentence in English. For instance, the word < سنذهب > /sanaᵭhab/ which comes from the three consonants’ root < ذهب > /ᵭhb/, is equal to the sentence "we will go" in English. Here, the root is affixed by the morphemes < ن /س > /s, n/, to determining the tense of the verb (< س > /s/, for the future) and the personal pronoun (i.e., subject) for the number and person (< ن > /n/, for “we”) (see Asadi et al., 2017a, 2017b). As for Arabic derivational morphology, words are produced from the combination of three-to four consonants roots, representing the meaning, and patterns representing the lexical and syntax categories (Saiegh-Haddad, 2013; Saiegh-Haddad & Henkin-Roitfarb, 2014). Arabic morphological transformations, contrary to other languages like English, are often nonlinear, disrupting the phonological and orthographic identity of words and reducing their morphological transparency (Levin et al., 2008; Saiegh-Haddad & Joshi, 2014).

### A Glimpse into Predictors of Reading and Reading Comprehension in Arabic

The review of reading and reading-related research on Arabic is beyond the scope of this brief overview. However, without attempting to provide an exhaustive summary of this literature, it is important to highlight that numerous studies have focused on the diglossic aspects of Arabic in reading-related research (Andria et al., 2022; Eviatar & Ibrahim, 2014; Joubran-Awadie & Shalhoub-Awwad, 2023; Khateb & Ibrahim, 2022; Rakhlin et al., 2021; Saiegh-Haddad, 2003, 2018; Saiegh-Haddad & Everatt, 2017; Saiegh-Haddad & Joshi, 2014; Saiegh-Haddad et al., 2020; Tarabya et al., 2021). Other studies focused on specific linguistic features, particularly orthographic (Abdelhadi et al., 2011; Khateb et al., 2013, 2014; Taha & Khateb, 2013) and morphological (Abu–Rabia, 2002; El Akiki & Content, 2020; Khateb et al., 2022; Saiegh‐Haddad & Taha, 2017; Tibi, 2016) during word recognition and reading processes (Abu–Rabia, 2002; Hassanein et al., 2023; Layes et al., 2015; Makhoul, 2017; Tibi & Kirby, 2018) but also during reading comprehension (Abu–Rabia, 2002; Asadi & Kasperski, 2024; Asadi et al., 2017a, 2017b; Bin Sawad et al., 2022; Mahfoudhi et al., 2010; Rakhlin et al., 2021; Saiegh-Haddad & Joshi, 2014; Schiff & Saiegh-Haddad, 2018; Taha, 2013; Taha & Saiegh-Haddad, 2017; Vaknin-Nusbaum & Saiegh-Haddad, 2020). In addition, an increasing number of studies had put more focus during the last years on predictors of reading development in children. To give only some examples, a longitudinal study among kindergarten to second grade Arabic-speaking children conducted by Abu Ahmad et al. (2014) found that the predictors of early reading rely heavily on sub-lexical and lexical abilities, which together explained 33% of the variance in second grade. The most significant predictors were phonemic awareness and phonological processing, visual orthographic processing and morphological knowledge. Layes et al. (2015)’s study investigated the potential contribution of cognitive reading-related skills to word reading among 4th and 5th grade Arabic-speaking children. They reported that PA and verbal working memory were significant predictors of word reading. In Tibi and Kirby (2018)’s study, the authors investigated the predictors of word reading among 201 Arabic-speaking third graders and showed that PA and naming speed were significant and unique predictors in reading accuracy and rate. In a more recent study, Hassanein et al. (2023) carried out a cross sectional study (1,098 children in grades 1 through 3) to examine the predictors of word reading (accuracy and rate). In brief, they found that, in first grade, phonological processing and orthographic processing were best predictors of reading accuracy, and together with morphological processing were also significant predictors of word reading fluency. In the second and third grade, RAN measures were found to explain unique variance in reading fluency (see also among first grade children Saiegh-Haddad, 2005). In another study, which included a large number of cognitive and linguistic and reading related (PA and RAN) measures, Asadi and colleages (2017)’s cross-sectional investigation examined the contribution of phonological, linguistic and cognitive variables to reading among a large sample of 1305 first to sixth grade Arabic-speaking children. Their results showed that while memory and orthographic knowledge consistently contributed to decoding and fluency, PA contributed mainly to decoding accuracy (see also Saiegh‐Haddad & Taha, 2017) up to the sixth grade, while RAN contributed mainly to fluency. In another study conducted among 458 first and second grade children, PA, vocabulary and RAN measures were shown to be significant predictors of reading in both grades, although the amount of explained variance was higher in first than in second grade (Asadi & Khateb, 2017). Taken together, these studies highlight the importance of both PA and RAN in predicting reading development in Arabic, as in many other languages, while also underscoring the role of additional linguistic (e.g., orthographic and morphological knowledge, vocabulary) and cognitive factors, particularly working memory.

In a similar manner, there has been also some increase in the number of studies examining the predictors of reading comprehension in Arabic (see Vaknin-Nusbaum & Saiegh-Haddad, 2020). Alrashidi (2010) examined the predictors of reading comprehension among 3 to 6 grades Arabic-speaking Kuwaiti children. The analysis compared the same predictors in regression results among grade 3 and 4 and among grade 5 and 6 and found that sound deletion and RAN were significant predictors of RC in the former while in the latter non-word reading and reading speed were also a significant predictor. In another study by Mahfoudhi et al. (2010), the authors examined 3rd and 6th grade Kuwaiti children and reported that phonological awareness and morphological knowledge measures (morphological segmentation task and morphological production task) contributed to reading comprehension beyond gender, grade and general ability. In a cross-sectional study that assessed validity of SVR among a large sample of Arabic speaking readers children, Asadi et al., (2017a, 2017b) examined first to sixth grade children and found that the basic components of the SVR (i.e., decoding and listening comprehension) explained between 38 and 56% of the variance in reading comprehension. The same study showed that inclusion of orthographic and morphological knowledge to an extended model explained up to 66% of the variance. Layes et al. (2015) investigated the potential contribution of cognitive and reading-related skills to reading comprehension among Arabic-speaking fourth and fifth grade readers. They found that PA and verbal working memory significantly explained variance in reading comprehension. In the study carried out by Vaknin-Nusbaum and Saiegh-Haddad (2020) among second graders, the authors reported that morphological awareness has an important role in predicting reading comprehension in Arabic, with awareness of derivations being a stronger predictor than inflectional awareness. In the same vain, a recent review by Bin Sawad et al. (2022) which included sixteen studies from across the Arab-speaking world and comprised readers from grade 1 to 12, the authors concluded that morphological awareness was the strongest predictor of reading comprehension, but also with PA being more associated with reading comprehension in the early grades. Finally, in a recent study, Asadi (2020) examined the predictors of reading comprehension among 7th and 9th graders. The findings indicated that, although PA and morphological knowledge were not significant predictors among typical readers (in contrast to readers with reading difficulties), vocabulary and syntactic knowledge contributed significantly to the variance in reading comprehension.

Previous research on reading and reading comprehension has primarily focused on school-aged children, leaving adult populations comparatively understudied. However, in recent years, there has been a growing body of research examining the predictors of reading and reading comprehension among adult students. Such studies aimed at examining whether early predictors of reading ability extensively described among children in various languages and orthographic systems (Earle & Del Tufo, 2021; Sucena et al., 2023) maintain their predictive power in adult readers. In this regard, various studies showed that a number of measures continue to play an important role in predicting reading among adults such as PA (Binder & Borecki, 2008; Binder et al., 2012; Greenberg et al., 1997; MacArthur et al., 2010; Mellard & Fall, 2012; Nanda et al., 2010; Thompkins & Binder, 2003), rapid naming (Eloranta et al., 2019; Greenberg et al., 2013; Swanson & Hsieh, 2009) and orthographic knowledge (Binder et al., 2012; Butler et al., 1984; MacArthur et al., 2010). As for studies assessing factors that predict reading comprehension among adults, several studies also demonstrated that predictors such as decoding (Landi, 2010; Mellard & Fall, 2012; Nanda et al., 2010), metalinguistic skills (Binder et al., 2012; Talwar et al., 2014; To et al., 2016), fluency (MacArthur et al., 2010; Mellard et al., 2010), oral language comprehension (Mellard & Fall, 2012; Sabatini et al., 2010), vocabulary (Braze et al., 2007; Carlisle, 2003; Hall et al., 2014; Landi, 2010; Tighe, 2012) and morphological knowledge (Earle & Del Tufo, 2021; Kotzer et al., 2021; Law et al., 2015; Maag, 2007; Metsala et al., 2019; To et al., 2016; Wilson-Fowler & Apel, 2015) maintain their predictive power among adults. However, with few exceptions, most findings on adult reading skills are derived from studies conducted in English and other European languages, with very limited evidence from Arabic. Because results from these studies cannot be generalized to Arabic (where reading develops within a complex orthographic system and a uniquely challenging diglossic context) the present study aimed to address the following research question:

To what extent do the same reading-related PA and RAN measures contribute to reading accuracy, speed and comprehension among Arabic-speaking adult students?

Based on previous studies on Arabic and other languages, in particular among adults, we hypothesized that:

#### H1

Phonological awareness and morphological knowledge will significantly predict word reading accuracy.

#### H2

Rapid automatized naming and morphological knowledge will significantly predict word reading rate, with RAN expected to emerge as the strongest predictor.

#### H3

Reading comprehension will be predicted by a combination of linguistic (phonological, morphological) and probably other cognitive factors.

## Material and Methods

### Participants

A total of 120 adult students, native speakers of Arabic, aged 18–32 years (*M* = 22.14, *SD* = 2.38), participated in this study (25 men, 95 women). Participants were recruited from 45 Arab or mixed localities in northern Israel and were enrolled in various academic disciplines (e.g., Education, Psychology, Computer Science). All participants were right-handed (Laterality Index: *M* = 79.9, *SD* = 28.3) as assessed by the Edinburgh Handedness Inventory, and were recruited from the University of Haifa and neighboring academic institutions.

### Procedure

Advertisements about the experiment were published on social media platforms and throughout the university campus. Those who reached to the experimenters were interviewed to exclude participants with known neurological or psychiatric illness. Individuals who satisfied the requirements to take part in the research were asked to provide an informed written consent for participation (as required by the Ethics committee for experiments in humans, Faculty of Education, University of Haifa, approval no’ 143/20). Before starting the testing session, all participants shared their demographic details and completed a hand dominance questionnaire. The testing session (~ 2 × 45 min sessions, with a short break) took place in a quiet room where each participant was individually examined with a battery of reading, linguistic and cognitive tasks.

### Research Tools

*Reading measures:* All participants underwent a word reading test and a reading comprehension test:

*Reading isolated words (*Asadi et al., 2014*):* Participants were instructed to read a list of words as accurately and fast as possible. This task included 40 partially short-vowelized words, 12 of which are verbs and 28 are nouns. The number of characters varied between 3 and 11 letters (average word length *M* = 6.25, *SD* = 1.53) and the number of syllables varied between 2 and 6 (Average syllable length *M* = 3.8, *SD* = 0.98(. Reading accuracy and speed were recorded. Reading rate was then computed as the number of words read by minute. The test reliability was α = .71 (see examples in Appendix 1).

*Reading comprehension:* In this test, participants were required to silently read a 533-word informational text, sourced from Israel’s Psychometric Entrance Test (PET, from October 2012, https://www.nite.org.il/psychometric-entrance-test/preparation/arabic-practice-tests/). The researchers created 13 questions that targeted four levels of comprehension: literal (N = 3), interpretive (N = 5), applied (N = 3), and affective (N = 2). These questions included both multiple-choice (N = 11) and open end questions (N = 2). Participants were instructed to read the text and complete the questions within 15 min. Response accuracy and total time (although not further analyzed) were measured.

*Reading-related measures*: All participants were administered two tests of phonological awareness (phoneme deletion and phonemic segmentation) and two tests of rapid automatized naming (RAN letters and digits):

*(i) Phonemic segmentation* (Asadi et al., 2014): In this 25-items test, the students’ ability to segment the words into their basic sounds was evaluated. The items represented 19 words (spoken Arabic (N = 7), literary Arabic (N = 12) and 6 pseudowords (see examples in Appendix 1). The number of phonemes varied between 2 and 6 (*M* = 3.88, *SD* = 1.18) and the number of syllables between 1 and 2 (*M* = 1.44, *SD* = 0.49). For each item, the participant had to repeat each word after the examiner and to segment it into its individual sounds. Segmentation accuracy was determined as the number of correct responses. The reliability of the test (α) was 0.79.

*(ii) Phonemic deletion *(Asadi et al., 2014): In this 20 items test, the students’ ability to delete a phoneme at the beginning, middle or end of words (11, 3, 6 items respectively) was examined. The number of phonemes in the words varied between 3 and 8 phonemes (*M* = 5.35, *SD* = 1.62, 1–3 syllables). Each word was read by the examiner to the participant who had to repeat it and to say it after deleting a specific sound (see examples in Appendix 1). Accuracy was determined as the number of correct responses. The reliability of the test (α) was 0.77.

*Rapid automatized naming*: This test was used to assess naming speed and included 2 subtests consisting each of 50 items (5 items repeated 10 times). The first subtest, RAN letters, included 5 Arabic letters: /s/, /a:/, /d̪ˁ/, /ʒ/, /θ/ (س/ا/ض/ج/ث /). The participants had to name all 50 items from right to left as fast as possible. The second test, RAN digits, included the 5 Arabic digits (1, 5, 9, 3, and 7). The participants had to name all 50 items from left to right. The naming time was measured for each sub-test.

*Linguistic measures:* These included two morphological knowledge tests (Words and verbs inflection) and two word fluency (semantic and phonological) tests:

*(i) Words and verbs inflection (*Asadi et al., 2014*)*: This test examined the students’ ability to inflect verbs and nouns in standard Arabic. In this task, participants were asked to produce the verb/noun verbally based on a root/name, according to gender, tense and number. This test included 33 items, the verb part included 7 roots, each root had a specific gender-number morpheme, and participants were asked to inflect each root- gender- number to three tenses (past, present and imperative), resulting in 21 items. In the name inflection part, there were three names which participants were asked to inflect according to four gender-number morphemes (using only possessive pronouns) resulting in 12 items. Accuracy was determined as the number of correctly given answers. The reliability of the test (α) was .78 (see examples in Appendix 1).

*(ii) Morphological fluency task* (Asadi et al., 2014): In this task, participants were asked to generate as many as possible words derived from a given three consonants in one minute. These roots were used: (i) (ج.ل.س) /g/ /l/ /s/; (ii) (ن.ظ.ف) /n/ /ðˁ/ /f/ and (iii) (ب.ر.د) /b/ /r/ /d/ ). The number of items generated from each root was recorded and the total number was calculated for the three roots (see examples in Appendix 1).

Two word-fluency test (referred to as "controlled oral word association"(Carone, 2007) were used to evaluate the spontaneous production of words beginning with a given letter (e.g., p) or a given category (animals) within a given time.

*(i) Semantic fluency test:* In this task the participants were required to orally produce, in one minute, as many as possible words corresponding to a given semantic category such as fruits and vegetables, animals and transportation means. Performance was determined by counting the number of correct words produced in 1 min for three different categories.

*(ii) Phonological fluency test:* In this task, the participants were required to generate orally words that begin with a specific sounds, usually B /ب/, Sh /ش/, and K /ك/. Repetitions and the same word with a different suffix were excluded from the count of correct responses. The total score of correct responses in each fluency letter task was recorded.

Executive Function Measures: *These measures included working memory, shifting (mental flexibility) and inhibition test:*

*The auditory forward and backward digit span tests (*Wechsler, 1998*):* These tests were administered to assess participants’ short term and working memory. Participants were asked to repeat a series of digits after hearing them from the experimenter who presented them at a rate of one digit/s, either in the same order (i.e., digit-span forward) or in the reverse order (i.e., digit-span backward). The maximal level to which the participants reached was determined for each sub-test.

*Color trail test *(D’Elia et al., 1989): This test assessed the participants' shifting abilities included two parts: Part 1 and Part 2. Both parts contain grapho-motor tasks requiring the use of pencil and paper. Color Trails 1 requires the participant to quickly and correctly sequence numbers from 1 to 25. All odd numbers (1,3,5,7….) are embedded within circles that have a pink background, while all even numbers (2,4,6,8…) are embedded within circles that have a yellow background. Scoring consists of time in seconds from initiation to completion of the task. The number of errors made were recorded. Color Trails 2 contains duplicates of each number from 1 to 25 embedded within pink and yellow circles. The participant was required to quickly connect the circles in ascending order, but alternating between pink and yellow colors. In other words, the participant would connect Pink 1 with Yellow 2 then to Pink 3 and so on through the number 25. Scoring consists of time in seconds from initiation to completion of the task. Response time for part 1 and part 2, together with the time difference between the two parts, computed as a "Switching/Shifting cost" measure, were used in the analysis.

*Stroop color-word test:* The Stroop Color-Word Test was used to assess participants' sensitivity to interferences and thus their ability to inhibit irrelevant information. The test used here was adapted to the Arabic language using the translation equivalents of the words in the Stroop 24 items test based on Stroop (1935). In the version used here, there were three parts of the test. In part 1, the Neutral Color Condition, the participants named the colors of circles printed in red, blue, green and yellow. In part 2, Congruent Word-Color Condition, the participants were asked to name the color of words of color names printed in the same color as the color name (e.g., the word red written in red). Finally in part 3, the Interference Word-Color Condition, the participants were asked name the color in which the words were presented, while ignoring the printed word itself which corresponds to another color name (e.g., the word red written in blue). This incongruence between the word’s color and identity required inhibiting the automatic reading of the word and selecting the appropriate response. Scoring consists of time in seconds from initiation to completion of each part. Response time for part 1 and part 3, together with the time difference between part 3 and part 1, as the "Stroop effect" measure, were used in the analysis.

*Auditory verbal learning and long term memory:* The Rey auditory verbal learning was used here to assess participants learning and long term memory capacities. In brief, the test which includes different sub-tests, consists of 15 unrelated common nouns, which in the first part are read to the participants, at the rate of one word per second and then followed by a free recall task (Sharoni & Natur, 2014). This step was repeated in five consecutive trials (Trials 1 through 5) and thus allows determining the individual learning curve (thought 1 to 5) and maximal learning ability at trial 5 (hereafter RAVLT Max). Twenty minutes later, and without an additional reading, participants were asked to recall the 15 words list (hereafter RAVLT DR).

#### Non-verbal General Ability

To control for the participants’ general non-verbal ability, the Raven Progressive Matrices test was administered (RPM, (Raven, 2003). This test consists of 60 items (matrices) where in each the participant is asked to choose from six simultaneously presented alternatives the missing part that completed a matrix. For the purpose of this study, two 30-items subtests of the RPM were designed by using in one the odd items and in the other the even test items. Correct answers were recorded for this task. Half of the participants were administered one subtest (odd items) and the other half the second subtests (i.e., even items). The test reliability α = .68.

### Data Analysis

Descriptive statistics were computed for all the measures collected. Inter-correlations were computed between all the study reading-related, linguistic and cognitive measures (predictors, independent variables). Pearson’s correlations were carried out to evaluate the relations between reading measures (i.e., dependent variables: word reading accuracy, word reading rate, and reading comprehension) and all independent variables. Separate stepwise regression analyses were then conducted to determine the contribution of each of the control and independent variables to word reading accuracy, word reading rate and to reading comprehension. SPSS v.27 was used for conducting all analyses presented here below (IBMCorp Ibm, 2017).

## Results

### Descriptive Statistics

Table 1 displays the descriptive analysis (means, standard deviations (SD) and ranges) among all participants. In these independent measures, the scores represent either the number of correct items (or in %), or the total time for each sub-test (in seconds: RAN, Stroop and Color Trail test). For the Semantic and phonological fluency task, values indicate the row score of correct answers and for the RAVLT task, the values indicate the number of items participants recalled (in the fifth trial and in delayed recall).

**Table 1:** Descriptive statistics (mean and SD) of the participants’ (*N* = 120) scores in the different measures

	Mean (SD)	Range	Skewness
General ability			
Raven	81.0 (11.0)	50	–0.98
Read. Related measures			
Phon. Seg	68.0 (20.1)	88	–0.59
Phon. Del	85.8 (14.9)	70	-1.51
RAN Letters	24.2 (4.9)	27	0.76
RAN Digits	17.8 (3.0)	13	0.30
Linguistics measures			
Morph. Flu	26.9 (8.6)	45	0.49
Morph. Inf	30.7 (3.4)	18	–2.21
Sem. Flu	42.0 (12.0)	71	0.92
Phon. Flu	28.5 (9.0)	51	0.93
Executive functions			
Digit (Fwd)	5.3 (0.9)	4	–0.05
Digit (Bwd)	4.1 (0.8)	4	0.33
CTT (A)	33.9 (11.3)	60	0.83
CTT (B)	74.3 (20.2)	100	1.11
CTT (B-A)	40.1 (15.9)	95	0.61
Stroop Neut	12.2 (2.4)	13	0.56
Stroop Inter	20.9 (5.6)	32	1.01
Stroop Effect	8.7 (5.4)	32	1.17
LTM			
RAVLT Max	13.2 (1.8)	8	–1.27
RAVLT DR	10.7 (2.8)	14	–0.48

*Phon. Seg.* for phonological segmentation*; Phon. Del.* for phonemic deletion; *RAN* for rapid automatized naming; *Morph. Flu.* for morphological fluency (normalized values); *Morph. Inf.* for morphological inflection; *Sem. Flu.* for semantic fluency; *Phon. Flu.* for phonological fluency; *Digit (Fwd)* for forward digit span; *Digit (Bwd)* for backward digit span; *CTT* for Color Trails Test; *Stroop Neut.* for stroop neutral condition; *Stroop Inter.* for Stroop interference condition; *Stroop Effect* for the difference between Inter. and Neut.; *RAVLT Max.* for Rey auditory-verbal learning test maximum learning; *RAVLT DR* for delayed recall. For the *RAN* test, values indicate total time in seconds to complete the task. For the *Phon. Seg.* and *Phon. Del.* the values indicate the percentage of accuracy percent. For the *Digit Span* (*Fwd* and *Bwd*), values indicate the number of final level participants reached. For the *Sem. and Phon. Flu.* task, values indicate the row score of correct answers. For the *CTT*, the values indicate the time in seconds. For the *RAVLT* measures, the values indicate the number of items participants recalled

To examine the extent to which the different linguistic measures were associated, inter-correlations were computed between all independent measures. Table 2 shows the coefficients only for the significant correlations between the different independent variables. As shown here, almost all significant correlations were weak to moderate (except those derived from related measures, see CTT B-A, Stroop effect = .83 to .90). Table 3 depicts the correlations between the dependent variables and the three independent variables, namely word reading accuracy, word reading rate and reading comprehension. Of note is the fact that the two phonological tasks (*r* = .60 and *r* = .64), in addition to morphological infliction (*r* = .40) showed the highest correlations with word reading accuracy. RAN digits and phonological fluency yielded the highest, although moderate, correlations with word reading rate (*r* = .37, *r* = .40, respectively). As for reading comprehension, together with correlations with some linguistic measures, it appeared that both Stroop interference and the RAVLT measures (learning and long-term memory measures) demonstrated also weak to moderate correlation (btween.30 to.35).

**Table 2:** Pearson correlations between all independent variables

Measure	1	2	3	4	5	6	7	8	9	10	11	12	13	14	15	16	17	18
1. Phon. Seg	1																	
2. Phon. Del	.51	1																
3. RAN Letters	.30	− .20*	1															
4. RAN Digits	.40		.60	1														
5. Morph. Flu	.21*				1													
6. Morph. Inf	.20*	.32			.33	1												
7. Sem. Flu	.29	.20*	− .31	− .21			1											
8. Phon. Flu	.30	.20*	− .26	− .42			.54	1										
9. Digit (Fwd)			.20*				− .33	.24*	1									
10. Digit (Bwd)	.21*	.22*			.31				.36	1								
11. CTT (A)			.70	.30			− .23*	-.26			1							
12. CTT (B)			.21*				− .30				.65	1						
13. CTT (B-A)												.83	1					
14. Stroop Neut	− .24*		.26	.28			− .40		.23*		.22*	.22*		1				
15. Stroop Inter	− .22*	− .24	.23*	.21*	− .26			− .19*		− .37	.40	.45	.29	.33	1			
16. Stroop Effect		− .21*			− .27	− .19*			− .21*	− .39	.32	.40	.27		.90	1		
17. RAVLT Max							.22*					− .23*	− .21*		− .31	− .30	1	
18. RAVLT DR	.25	.20*	.25	− .20*			.40	.31				− .30	− .25		− .36	− 0.30	.70	1

All missing cells refer to non significant correlations, values without * are all significant at least at p < 0.01, and values with * are all significant at p < 0.05. See all abbreviation in Table 1

**Table 3:** Correlations between the dependent variables and all linguistic and cognitive measures

	Word reading ACC	Word reading rate	Reading comprehension
	*M* = 88.3 (*SD* = 8.40)	*M* = 41.9 (*SD* = 13.23)	*M* = 83.8 (*SD* = 15.87)
Age	.11	− .13	.01
Raven	.02	− .10	.21*
Phon. Seg	.64**	.33**	.34**
Phon. Del	.60**	.29**	.31**
RAN letters	− .24*	− .21*	− .12
RAN digits	− .24**	− .36**	− .17
Morph. Flu	.24**	.23*	.25**
Morph. Inf	.39**	.23*	.15
Sem. Flu	.16	.26**	.10
Phon. Flu	.26**	.39**	.13
Digit (Fwd)	.07	− .04	.19*
Digit (Bwd)	.18	− .01	.16
CTT (A)	− .06	− .26**	− .15
CTT (B)	.03	− .21*	− .18
CTT (B− A)	.08	− .08	− .13
Stroop Neut	.09	− .14	− .04
Stroop inter	− .15	− .16	− .34**
Stroop effect	− .15	− .11	− .34**
RAVLT max	.07	.06	.28**
RAVLT DR	.16	.12	.35**

See all abbreviation in Table 1. * for *p* < .05; ** for *p* < .01

### Regression Analyses

In order to assess the predictive power of each of the study independent variables, regression analyses were conducted separately for word reading accuracy, word reading rate and reading comprehension. A stepwise procedure was used to sequentially include the predictors, based on their contributions to the variance in the dependent variable. Stepwise regression involves adding independent variables one at a time and conducting an *F*-test for each variable included in the model. This process continues until no new significant independent variables can be added, and none of the insignificant independent variables can be removed from the regression equation. In the analysis reported hereafter, we entered age and the Raven’s general non-verbal ability measures as a control variables, then we included all other variables in four different steps as follows: (i) Reading related measures (phonological segmentation, phonological deletion, RAN letters and RAN digits), (ii) Linguistics measures (morphological fluency, morphological inflection, semantic and phonological fluency), (iii) Executive functions’ measures (forward and backward digit span, Color Trails measures, and Stroop measures), and (iv) the RAVLT’s measures) as detailed in Table 1. The results of three separate stepwise regressions, conducted to determine the contribution of exactly the same predictors to word reading accuracy, reading rate and reading comprehension, are presented successively in Tables 4, 5 and 6.

*Word reading accuracy*: Table 4 displays the results of the stepwise regression analysis for word reading accuracy. It shows that the model explained 55% of the variance (*R*^*2*^ = .55; *F* (1,112) = 8.8; *p* < 0.005) in reading accuracy. In the first model, age and Raven did not significantly explain variance. In the subsequent models, only three predictors were statistically significant: phonological awareness (phonemic deletion and segmentation) and morphological Inflection. The regression coefficients showed that the highest weight of contribution was for phonemic segmentation, which in model 2 explained ~ 42% of the variance (β = .63, *p* < .00), reflecting a large practical effect, followed by phonemic deletion in model 3 (β = .37, *p* < .00) which added 10% to the variance and together explained 52%. Finally, model 4 added the morphological inflection measures (β = .20, *p* < .00) which added 3% more explained variance for up to a total of 55%. The overall model yielded a large effect size (Cohen’s ${f}^{2}=\sim 1.22$), indicating strong practical explanatory power for reading accuracy. These results suggest that reading accuracy relies on both phonological decoding and morphological knowledge.

**Table 4:** Step-wise regression analysis for word reading accuracy

Measures	*β*	*t*	*P*	*R*^*2*^
Model 1				− 0.01
Age	0.13	1.35	0.18	
Raven	0.01	0.16	0.87	
Model 2				0.42
Age	0.09	1.36	0.18	
Raven	− 0.01	− 0.18	0.86	
Phon. Seg	0.63	8.83	**0.00**	
Model 3				0.52
Age	0.10	1.50	0.14	
Raven	− 0.04	− 0.65	0.52	
Phon. Seg	0.45	5.84	**0.00**	
Phon. Del	0.37	4.87	**0.00**	
Model 4				0.55
Age	0.10	1.68	0.09	
Raven	− 0.04	− 0.70	0.50	
Phon. Seg	0.44	6.00	**0.00**	
Phon. Del	0.31	4.10	**0.00**	
Morph. Inf	0.20	2.30	**0.00**	
Model Summary				
R^2^	0.55		**0.00**	
Adjusted R^2^	0.53			
F-Statistic	8.80			

See all abbreviation in Table 1. The *β* values presented here are standardized coefficients. Bold values are significant values

*Reading rate*: The stepwise regression analysis conducted to test the contribution of the same predictors to word reading rate showed that the model was significant (*R*^*2*^ = .32; *F* (1,111) = 5.04; *p* < 0.05) and explained a total of 32% of the variance, while controlling for age and Raven in the first step (see Table 5). Afterwards, the regression coefficients showed that the RAN digits (*β* = -.37, *p* < .00) was the major predictor of reading rate and explained 16% of the variance in model 2 reflecting a moderate to large effect and confirming its central role in fluency, then in model 3, phonological deletion (β = .25, *p* < .00) added 6% more to the explained variance for a total of 22%. In model 4, phonological fluency (β = .19, *p* < .00) also added 6% to the explained variance, and finally in model 5, morphological fluency (*β* = .18, *p* < .00) added 4% more of explained variance up to 32%. The regression model showed a medium to large effect size (Cohen’s ${f}^{2}=\sim 0.47$), suggesting a meaningful practical contribution of the predictors to reading rate. Overall, together with RAN digits which emerged as the strongest predictor, phonological deletion, phonological fluency, and morphological fluency contributed additional variance, indicating that reading speed reflects both rapid naming efficiency and broader linguistic retrieval skills.

**Table 5:** Stepwise multiple regression analysis for word reading rate

Measures	*β*	*t*	*P*	*R*^*2*^
Model 1				0.03
Age	− 0.13	− 1.42	0.15	
Raven	− 0.09	− 1.08	0.28	
Model 2				0.16
Age	− 0.14	− 1.63	0.10	
Raven	− 0.11	− 1.26	0.20	
RAN Digits	− 0.37	− 4.28	**0.00**	
Model 3				0.22
Age	− 0.14	− 1.74	0.08	
Raven	− 0.13	− 1.60	0.11	
RAN digits	− 0.33	− 3.92	**0.00**	
Phon. Del	0.25	3.01	**0.00**	
Model 4				0.28
Age	− 0.12	− 1.5	**0.04**	
Raven	− 0.16	− 2.00	0.13	
RAN digits	− 0.21	− 2.48	**0.01**	
Phon. Del	0.22	2.70	**0.00**	
Phon. Flu	0.19	2.31	**0.02**	
Model 5				0.32
Age	− 0.16	− 2.10	**0.04**	
Raven	− 0.14	− 1.8	0.07	
RAN digits	− 0.22	− 2.50	**0.01**	
Phon. Del	0.20	2.50	**0.01**	
Phon. Flu	0.27	3.10	**0.02**	
Morph. Flu	0.18	2.25	**0.02**	
Model summary				
R^2^	0.32			
Adjusted R^2^	0.28			
F-Statistic	5.04		0.02	

See all abbreviation in Table 1. The *β* values presented here are standardized coefficients. Bold values are significant values

*Reading comprehension*: The third stepwise regression analysis conducted on reading comprehension showed that the model explained 28% of the variance (*R*^*2*^ = .28; *F* (1,111) = 9.54; *p* < 0.005, see Table 6). In the first model which explained 4% of the variance, Raven appeared as a significant predictor. In model 2, the addition of phoneme deletion raised the explained variance to 14%. In model 3 morphological fluency contributed an additional 4% of explained variance. Also 4% more of explained variance were added in model 4 owing to the Stroop effect measure. Finally, in model 5, the RAVLT’s delayed recall measure, indexing long term memory, added 6% more to the explained variance for a total of 28%. Notably, the Stroop effect no longer reached statistical significance in Model 5 (*p* = .12) following the inclusion of the delayed recall measure, suggesting that its contribution may be partially mediated by memory-related processes. The model demonstrated a medium effect size (Cohen’s ${f}^{2}=\sim 0.39$), indicating a moderate but practically relevant level of explained variance in reading comprehension. Overall, the results highlighted the multidimensional nature of reading comprehension as it predicted by a combination of linguistic and cognitive factors including general non-verbal ability, phonemic deletion, morphological fluency, response inhibition and long-term memory (delayed recall).

**Table 6:** Stepwise multiple regression analysis for reading comprehension

Measures	*β*	*t*	*P*	*R*^*2*^
Model 1				0.04
Age	0.04	0.44	0.65	
Raven	.021	2.30	**0.02**	
Model 2				0.14
Age	0.03	0.39	0.70	
Raven	0.18	2.04	**0.04**	
Phon. Del	0.31	3.52	**0.00**	
Model 3				0.18
Age	0.03	0.34	0.74	
Raven	0.16	1.82	0.07	
Phon. Del	0.28	3.30	**0.00**	
Morph. Flu	0.20	2.40	**0.01**	
Model 4				0.22
Age	0.06	0.70	0.50	
Raven	0.13	1.51	0.13	
Phon. Del	0.25	2.90	**0.00**	
Morph. Flu	0.15	1.74	0.08	
Stroop effect	− 0.22	− 2.43	**0.01**	
Model 5				0.29
Age	0.07	0.92	0.36	
Raven	0.09	1.10	0.30	
Phon. del	0.20	2.50	**0.01**	
Morph. flu	0.20	2.30	**0.02**	
Stroop effect	− 0.14	− 1.60	0.12	
RAVLT DR	0.27	3.08	**0.00**	
Model summary				
R^2^	0.29			
Adjusted R^2^	0.25		**0.00**	
F-Statistic	9.54			

See all abbreviation in Table 1. The *β* values presented here are standardized coefficients. Bold values are significant values

## Discussion

Extensive research has over the years investigated early predictors of reading and reading comprehension in school-aged children across diverse languages and orthographies. More recently, scholars have increasingly focused on whether these predictors remain relevant for adult reading skills. However, most of these studies (see Introduction) have been conducted primarily on English and other European languages, limiting the applicability of their findings to other contexts, especially those as challenging as in the diglossic Arabic linguistic situation. To address this gap, the present exploratory study aimed at investigating the role of linguistic and cognitive factors in reading skills among Arabic-speaking adult students. For this purpose, word reading, reading comprehension, and other reading-related linguistic and cognitive tasks were administered to a sample of 120 Arabic-speaking students, with their results analyzed using correlations and stepwise regression models.

The correlation analysis first conducted on the study independent variables showed that many significant, although weak to moderate, correlations were found between reading-related, linguistic and cognitive measures. In particular, the highest correlations were found within PA (*r* = . 51) and within RAN (*r* = . 60) measures, but also between other sub-tests of the same domain such as between semantic and phonological fluency tests (*r* = . 54), the Color Trail Part 1 and 2 time measures (*r* = . 65), or RAVLT MAX and DR sub-measures (*r* = .70). Concerning the correlations between the different independent and dependent variables, it is worth mentioning that the highest and most significant were found between PA (up to *r* = .64) and linguistic measures (in particular morphological inflection) and between these same variables (although more moderately) and reading rate. As for reading comprehension, the results clearly indicated that it also correlated with different measures of EFs and verbal long-term memory. As for the results of the regression analyses that will be discussed hereafter, it is worth noting that the same reading-related, linguistic and cognitive abilities differentially explained variance in reading accuracy, reading rate and reading comprehension up to 55%, 32% and 29% respectively. Importantly, consideration of standardized coefficients and effect size estimates further indicated that these predictors exerted small-to-moderate practical influences on adult reading performance beyond mere statistical significance. These percentages indicate that many other variables that contribute to the variance in reading abilities among adult university students were not considered in this study. Future studies among adult populations should mandatorily consider other linguistic (e.g., orthographic knowledge, vocabulary, syntax, etc.., (see for example Binder et al., 2017; Brimo et al., 2017; Dong et al., 2020; Nassaji & Geva, 1999; Ouellette, 2006)) and cognitive (e.g., visual processing, attention) domains but also more psychosocial factors (e.g., SES, reading self-concept, reading anxiety, attitudes towards Arabic, etc.., (see for example Chow et al., 2021; Courtenay-Brown, 1992; Katzir et al., 2009; Li et al., 2023; Soares et al., 2023)).

### Predictors of Reading

The first two analyses sought to examine which of the study independent variables contribute to reading accuracy and reading rate. In the stepwise regression analyses conducted to address this question, students’ age and their performance in the Raven Standard Progressive Matrices test (administered as a measure of their general non-verbal intellectual ability) were introduced in the first step as control variables. These analyses showed that control variables failed to significantly explain variance both in reading accuracy and rate regression models.

The results indicated that the contribution of reading-related PA and RAN skills to reading is consistent with findings from previous studies in other languages (Goswami et al., 2005; Ziegler et al., 2010) as well as with research conducted among Arabic-speaking children (Al Otaiba & Fuchs, 2002; Beidas et al., 2013; Chan & Wade‐Woolley, 2018; Shany & Share, 2011). In the largest cross-sectional study reported by Asadi et al., (2017a, 2017b), PA but not RAN, consistently predicted reading accuracy among children in Grades 1–6, whereas RAN consistently predicted reading fluency, with PA contributing only inconsistently and to a limited extent. In the present study, both PA tasks, phonemic segmentation followed by phonemic deletion emerged as the primary predictors of reading accuracy, whereas RAN was the strongest predictor of reading rate, followed by phonemic deletion.

The results presented here are in accordance with previous literature indicating that phonology plays a distinct role in reading among children (Abu-Rabia et al., 2003; Caravolas et al., 2005; Mann & Wimmer, 2002) and continues throughout adolescence and into adulthood (Blau et al., 2009; Cheema et al., 2023; Meade, 2020; Nergård‐Nilssen & Hulme, 2014; Stanovich & West, 1989). These results are in line with previous studies in Arabic where PA significantly predict reading accuracy from very early up to grade 10 (Asadi & Abu-Rabia, 2019; Asadi et al., 2017a, 2017b; Gottardo et al., 2020; Schiff & Saiegh-Haddad, 2018). The findings presented here extend previous work by providing evidence that phonological skills play a key role in adult reading and challenge the claim that the effects of phonology in transparent orthographies are transient and more prominent in lower than in higher grades (Goswami et al., 2005; Shatil & Share, 2003; Ziegler et al., 2010). The contribution of PA here may seem counterintuitive, in view of the fact that adult Arabic readers commonly use the opaque orthographic version (non-short vowelized) of their writing system, where one can expect more dependence on the lexical route (Coltheart et al., 2001). Hence, the involvement of PA in reading accuracy might have been driven by the fact that partially vowelized words (composed primarily of letter strings with typical spelling-to-sound correspondences) were used here (Asadi et al., 2023a, 2023b). A similar explanation has previously been advanced to explain the effect of PA in late primary school grades (Asadi et al., 2017a, 2017b). However, a previous study have shown that PA contributed similarly to reading both vowelized and unvowelized words both in very early (1st and 2nd, see Asadi & Khateb, 2017) and late (4th and 5th, see Asadi et al., 2023a, 2023b) elementary school grades. Thus, it is likely to assume that both sub-lexical and lexical processing routes could be used to identify these words among adult skilled readers while it is well established that these pathways do operate in a parallel way that is not independent (Paap & Noel, 1989).

Together with PA two measures, the morphological inflection measure was shown to significantly contribute to the explained variance (up to a total of 55%) in word reading accuracy. This finding aligns with many previous studies which emphasize the role of morphological knowledge to reading accuracy (Deacon & Kirby, 2004; Rothou & Padeliadu, 2015; Royle et al., 2019) including in Arabic in early and late school grades (Asadi et al., 2017a, 2017b; Saiegh-Haddad & Geva, 2008; Schiff & Saiegh-Haddad, 2018; Taha & Saiegh-Haddad, 2017). A recent study by Metsala et al. (2019) carried on 220 first year university students revealed that morphological awareness add unique contributions to students’ reading achievements beyond that accounted for by phonological awareness and orthographic processing. The role played by morphological knowledge is in line with Bar-Kochva (2016)’s results showing that morphological training among Hebrew-speaking university students with dyslexia induced positive effects on reading and spelling. Also, these findings lend support to models of reading which propose that morphological awareness exerts a direct influence on both word reading and reading comprehension (Perfetti & Stafura, 2014).

Among the cognitive predictors of reading rate, rapid automatized naming (RAN) have been the most extensively studied (Bentin et al., 1999; Clarke et al., 2005; Hornung et al., 2017; Logan et al., 2011; Manis et al., 1999; McBride-Chang & Manis, 1996; Norton & Wolf, 2012; Stanley et al., 2018). RAN has been found to be closely related to reading fluency in childhood (Georgiou et al., 2009; Moll et al., 2009; Papadopoulos et al., 2016) and in adulthood (Vukovic et al., 2004). Deficits in the RAN, as a domain-specific speed of processing measure, have been shown to be an independent risk factor for dyslexia across various languages (Beidas et al., 2013; Breznitz & Meyler, 2003; Catts et al., 2002; Fawcett & Nicolson, 1994; Fawcett et al., 1996; Law et al., 2015; Pennington et al., 2001; Sobotka & May, 1977; Stanovich, 2000; Willcutt et al., 2005; Wolf & Bowers, 1999). Childhood RAN skills have been found to predict developmental dyslexia (Landerl et al., 2013). Torppa et al. (2015) for example found that early problems in RAN differentiated a group of children that had persistent dyslexia in adolescence from children with no later dyslexia. In a study by Eloranta et al. (2019) that investigated Finnish reading-disabled adult, diagnosed in childhood, the authors showed that, although reading disabled group globally showed poorer performance than controls, childhood rapid naming ability was a significant factor that differentiated those with persisting reading disability from those who have significantly improved their reading fluency, to a level that no longer met adult dyslexia criteria. Also, the authors suggested that the only major difference in cognitive skills between skilled and dyslexic readers was on RAN. Other studies in adults with dyslexia have also suggested that RAN deficit might become even more prominent than residual deficits in other core cognitive skills (Fernandes et al., 2017; Swanson & Hsieh, 2009).

Here we showed that contrary to reading accuracy where the RAN did not explain significant variance, RAN digits measure was the major predictor of reading rate and alone significantly contributed to about 13% of the variance. The observation here that RAN is more strongly related to reading speed than reading accuracy is in line with previous literature (see for a review Norton & Wolf, 2012; Papadopoulos et al., 2016; Siddaiah & Padakannaya, 2015). This finding particularly reflects the strong relation of RAN with reading speed\rate established in earlier studies (e.g., Heikkilä, 2015; Torppa et al., 2015; Vukovic et al., 2004) including in Arabic (Asadi & Shany, 2018; Asadi et al., 2017a, 2017b; Gharaibeh et al., 2021; Jabbour-Danial et al., 2024; Shany et al., 2023). In Araújo et al. (2015)’s meta-analysis which examined a total of 137 studies, the authors confirmed that RAN was more strongly related to reading fluency (where accuracy and speed are weighted), a finding that might be explained by the fact that both measures share time component. Indeed, other authors have reported that performance in RAN is highly associated with general processing speed (Catts et al., 2002; Georgiou et al., 2009; Papadopoulos et al., 2016). However, Araújo et al. (2015) claimed that, invoking processing speed as the single explanation for the correlation between RAN and reading is insufficient, since in this case a significant relationship with reading accuracy would not have been apparent, as observed here. In line with this conclusion, we found in our analysis that both RAN letters and RAN digits correlated, although weakly, with reading accuracy, but significant contribution for RAN was observed only in reading rate, which is a pure measure of rapid speed.

Our analysis showed that in addition to RAN, the most important predictor of reading rate was phoneme deletion task. In line with our results, a recent study carried by Memisevic et al. (2022) revealed that RAN and phoneme deletion task were the best predictor of text passage reading and word list reading tasks. The importance of PA in reading speed both in consistent and inconsistent orthographies also has long been established both in alphabetical (Caravolas et al., 2005; Ziegler et al., 2010) and non-alphabetical systems and such as Chinese (Newman et al., 2011). PA was shown to contribute, although non-consistently, to reading fluency among Arabic-speaking children (Asadi et al., 2017a, 2017b).

Consistent with previous observations (Kavé & Sapir-Yogev, 2023; Shareef et al., 2019), our results also demonstrated a significant contribution of both phonological and morphological fluency tasks. A large number of studies have focused on phonological\letter (and semantic) fluency, which are the most commonly used verbal fluency tasks in research reading among children (Brosnan et al., 2002; Kavé & Sapir-Yogev, 2023; Lipowska et al., 2008; Plaza et al., 2002) and adults (Hatcher et al., 2002; Mielnik et al., 2015; Shareef et al., 2019; Smith‐Spark et al., 2017). In the current study phonological fluency added ~ 7% to the explained variance in reading speed while morphological fluency added 3% more. These findings are consistent with the well-documented role of morphological knowledge (MA) in reading performance (Deacon & Kirby, 2004; Giazitzidou & Padeliadu, 2022; Giazitzidou et al., 2024; Hassanein et al., 2023; Kirby & Bowers, 2017; Nagy et al., 2006). In Asadi et al., (2017a, 2017b)’s study the contribution of MA to reading fluency was inconsistent among Arabic-speaking children in Grades 1–6. This limited effect may be attributable to the lack of specificity in the measurement, as MA was assessed using a composite score averaging performance across four different tasks. In contrast, subsequent studies have provided clearer evidence for the role of morphology in reading fluency. For example, Schiff and Saiegh-Haddad (2018) reported significant contributions of morphological knowledge among Arabic readers in Grades 2–10, and Vaknin-Nusbaum (2021) demonstrated that a MA intervention improved reading fluency among Hebrew-speaking elementary school children. More recently, Asadi et al. (2023) reported that knowledge of both derivational and inflectional morphology differentiated typical and poor readings in terms of reading fluency.

To sum up, the analysis reported here showed that these variables accounted altogether for 32% of the variance in reading rate, a finding that calls for future studies that include other important factors, in particular vocabulary and orthographic knowledge.

## Predictors of Reading Comprehension

The analysis reported in this study showed that a combination of measures differentially contributed to reading comprehension with first the reading-related phonemic deletion scores explaining about 10% of the variance beyond the Raven general ability (4%). The analysis showed that among the linguistic factors, only morphological fluency added significant explained variance. Of the cognitive factors, it was observed that the Stroop effect, that indexes individual sensitivity to interference negatively correlated with RC (i.e., the less one is sensitive to interference the higher is RC performance), explained also an additional 4% of variance. The final model showed that including memory measures to the analysis revealed that long-term delayed recall measure was a significant predictor adding 7% more of explained variance.

The fact that phonemic deletion, which was a steady predictor in both reading accuracy and rate, also explained in reading comprehension is an interesting finding that needs to be further discussed. In addition to the extensive previous research on the role of phonological awareness in word reading in various languages and orthographies (see Introduction), there are other studies, although less abundant, that assessed its relation with reading comprehension (Baker et al., 2022; Cárnio et al., 2017; Engen & Høien, 2002; Share & Leikin, 2004) including in Arabic (Asadi, 2020). In fact, a multitude of studies have examined the validity of the simple view of reading model (Gough & Tunmer, 1986; Hoover & Gough, 1990) in various languages and focused more particularly on the contribution of its basic components (i.e., decoding and listening comprehension) and less on other linguistic skills. These components, which were shown to account for approximately 40–80% of the variance in reading comprehension for readers ranging from 2nd through 10th grade among English speaking children (see review in Joshi, 2018) have been shown to explain up to 56% of the variance among Arabic-speaking elementary school children (Asadi et al., 2017a, 2017b). In a study conducted by Engen and Høien (2002) among a large sample of Norwegian first graders, the authors reported that PA contributed both directly and indirectly (through decoding) to the variance in RC. These findings are in consonance with our finding since we found that phonological deletion could explain 14% of the variance. In Arabic, Asadi (2020)’s study which examined the contribution of linguistic measures to RC among middle school students showed that PA explained variance in RC only among 7th grade reading disabled participants. In a very recent study by Saiegh‐Haddad and Schiff (2025), the authors examined Arabic native-speaking third graders with receptive vocabulary, phonological awareness, morphological awareness, and word decoding and reported that PA did not emerge as a significant predictor of RC. In a recent meta-analysis carried out by Baker et al. (2022) on 33 studies that examined relation between the essential components of reading and reading comprehension among Spanish children, the authors found that the largest effect sizes were between phonological awareness and reading comprehension, and between fluency and reading comprehension. The fact that previous literature suggests that PA might contribute to RC the literature, either directly or indirectly through decoding, emphasizes the need for this issue to be more systematically addressed in future studies. In fact, this study shows, as others in the literature, including those using the basic components of the SVR have generally shown, that a considerable amount of variance has not been explained the study factors. To give one flagrant example, Asadi et al., (2017a, 2017b)’s cross-sectional study that assessed the validity of the SVR among Arabic-speaking first to sixth grade children found that decoding and listening comprehension explained between 56 and 38% of the variance in RC. The inclusion of orthographic and morphological knowledge in the path analysis models accounted for an additional 10–22% of the variance. Very interestingly, the results of these extended models showed that decoding was no more significant among all grades and the contribution of listening comprehension, although significant all the way, became smaller. Future studies thus need not only include other factors but try to better disentangle the effects of each variable.

In addition to the role of phonological awareness, our findings indicate that morphological fluency, along with other factors, contributes to variance in reading comprehension. This finding is in line with previous studies in different languages (Carlisle, 2000; Deacon & Kirby, 2004; Deacon et al., 2014; Nagy et al., 2006; Verhoeven & Carlisle, 2006), including the Semitic Hebrew and Arabic languages (Abu-Rabia, 2007; Asadi, 2020; Asadi et al., 2017a, 2017b; Primor et al., 2011; Vaknin-Nusbaum, 2018; Vaknin-Nusbaum & Saiegh-Haddad, 2020). For instance, in a recent study among Hebrew among second and third graders, it was reported that morphological knowledge, which differentiated between children with good and poor reading abilities, contributed significance variance to reading comprehension (Vaknin-Nusbaum, 2018). A recent study by Asadi (2020) conducted among 7 and 9 grade Arabic-speaking good and poor readers found that morphological knowledge contribution to reading comprehension was more consistent among poor than the typical readers. Authors suggest that the contribution of morphological knowledge to reading comprehension increases (Berninger et al., 2010; Nagy et al., 2006) and reported that higher scores in reading comprehension among university students (when compared to less skilled readers) was associated with greater morphological awareness. Also, To et al. (2016) found that morphological awareness was a significant predictor of reading comprehension among two groups of adult college students with low literacy skills and skilled readers. In the same line, Wilson-Fowler and Apel (2015) showed that morphological awareness was a significant predictor of reading comprehension among undergraduate college students. Similar results were reported by Kotzer et al. (2021) amongst 71 undergraduate students.

Most of reading comprehension models (discussed in the introduction) suggest several linguistic, psycho-sociological and cognitive factors, but no emphasis had been put on executive functions. However, some relatively recent researchers have garnered support for the contributions of executive functions to reading comprehension above and beyond the skills of decoding and listening comprehension (Arrington et al., 2014; Cutting et al., 2009; Kieffer et al., 2013; Locascio et al., 2010). In our study, we found that both inhibition and long-term memory contribute to reading comprehension. These finding are in line a recent study by Nouwens et al. (2021) that examined fourth graders and found that executive functions, namely, inhibition contribute to reading comprehension. In another study by Arrington et al. (2014), the authors examined the relations of reading comprehension, decoding, working memory, and attentional control among 1,134 adolescents. Path analyses used to assess the direct and indirect effects of working memory and aspects of attentional control on reading comprehension and decoding showed significant direct effects of working memory, sustained attention, and cognitive inhibition. Moreover, a recent meta-analytic review carried by Follmer (2018) revealed moderate positive association between executive function and reading comprehension. Contrary to many previous reports in the literature reporting a relation between short term and working memory with reading comprehension (Arrington et al., 2014; Carretti et al., 2009; Goff et al., 2005; Masson & Miller, 1983; Talwar et al., 2018), short term and working memory (both verbal and visuo-spatial) measures did not contribute here to reading comprehension. Instead, the results showed that, despite the fact that little evidence has been provided regarding the role of long term memory in reading comprehension (Was & Woltz, 2007), delayed recall significantly contributed to reading comprehension and explained an additional 7% of the variance well beyond general ability, linguistic variables and cognitive inhibition. This finding might probably explain the absence of effect for verbal short-term memory (Majerus & D’Argembeau, 2011), thus calling for future studies to examine its meditating effects.

Importantly, the present findings extend the adult reading literature by providing evidence that the core predictors of reading identified in children remain differentially relevant in Arabic-speaking university students. In line with our hypotheses, phonological awareness emerged as the strongest predictor of reading accuracy, whereas rapid automatized naming primarily accounted for individual differences in reading rate, supporting theoretical accounts that distinguish between accuracy-based and speed-based mechanisms in skilled/unskilled reading (Shany et al., 2023; Wolf & Bowers, 1999). Moreover, the results contribute to novel evidence that reading comprehension in Arabic-speaking adults is not explained solely by linguistic skills but rather reflects an interaction between linguistic processes and higher-order cognitive control and memory resources, as reflected in the contribution of inhibition and long-term memory. These findings are theoretically meaningful because they suggest that even among skilled university adult readers, reading remains constrained by multiple interacting components, thereby supporting multifactorial frameworks of reading rather than single-deficit accounts.

Taken together, these findings suggest that although cognitive and linguistic skills contribute to reading and reading comprehension, they are unlikely to fully account for the variability observed in adult reading performance, thereby supporting the need to consider broader integrative frameworks. From a multifactorial perspective on reading development and proficiency, cognitive and linguistic skills constitute necessary, but not sufficient, conditions for explaining individual differences in adult reading outcomes. Contemporary models of reading, including extended versions of the SVR and componential frameworks, increasingly emphasize the interaction between cognitive-linguistic abilities and socio-psychological factors such as socioeconomic background, reading motivation, self-concept, anxiety, and attitudes toward literacy (Gough & Tunmer, 1986; Hoover & Gough, 1990; Katzir et al., 2009; Sümer Dodur & Ceylan, 2025). Although these factors were not measured in the present study, they may modulate the efficiency with which core skills such as phonological awareness, rapid automatized naming, and morphological knowledge are recruited during reading, particularly in adulthood where reading behavior is shaped by long-term educational experiences and affective engagement with text. In diglossic contexts such as Arabic, attitudes toward the LA and differential exposure to academic registers may further interact with cognitive predictors, potentially amplifying or attenuating their observable effects (Chow et al., 2021; Soares et al., 2023). The absence of these socio-psychological variables may therefore partially account for the unexplained variance observed in the regression models, especially for reading rate and comprehension.

## Conclusions and Limitations

To the best of our knowledge, few studies have assessed predictors of reading and reading comprehension among populations up to the age of 15 among Arabic-speaking participants. Also, to date few (if any) studies have investigated the predictors of reading abilities among skilled Arabic-speaking adults. In this regard, this exploratory analysis is the first of its kind to provide new and precious insights into the field of reading in Arabic. The results provide, although partial, some answers to the question of whether (and which) linguistic and cognitive skills that explain reading and reading comprehension in childhood maintain any prediction power in adulthood. This study clearly indicated that, among the variables examined here, reading-related (phonology and RAN) and linguistic variables continue to significantly but differentially predict word reading accuracy and speed, as in childhood. Our analysis showed that a relatively small amount of variance, particularly in reading rate (but also to some extent in accuracy), has been explained by the study predictors. This observation indicate that future studies should use other additional (linguistic, cognitive and psycho-social) variables, and other analysis approaches (such as hierarchical regression or structural equation modeling). In fact, given the known limitations of stepwise procedures (e.g., inflating type I errors, model instability and potential overfitting), findings should be interpreted cautiously and require replication in independent samples. Other approaches will mandatorily necessitate a larger sample size to investigate both direct and indirect contributions of such variables. As for reading comprehension in adult university students, the study also showed that a small amount of variance was explained here (with the linguistic variables explaining only 18%). This finding calls for future investigations to include not only other linguistic variables (such as listening comprehension, orthographic, syntactic and vocabulary measures) but also cognitive, psychological variables while examining the contribution of different types of texts. Indeed, an additional limitation of this study is the lack of socio-psychological indicators (e.g., SES, motivation, literacy attitudes), whose interaction with cognitive–linguistic skills may have contributed to the unexplained variance in the present models. In addition, the geographic and sociolinguistic characteristics of the present sample impose further constraints on the generalizability of the findings. All participants were Arabic-speaking university students recruited from northern Israel, a context that is characterized by specific educational, cultural, and sociolinguistic conditions that may not fully represent Arabic-speaking populations in other regions. Therefore, the present findings should be interpreted within the specific societal context of the studied population. Future research should replicate the current design in diverse Arabic-speaking communities across different geographic regions and educational systems to assess the robustness and boundary conditions of the reported results. Here again, a larger sample size will allow assessing different types of analysis to examine different groups of readers and comprehenders.

## Data Availability

The data that support the findings of this study can be found on OSF (https://osf.io/7xry5/overview).

## Appendix

Examples of the different stimulus in the different tasks in International Phonetic Alphabet (IPA).

**Table:**

Task\Example	English translation	International phonetic alphabet	Number of syllables
Reading isolated words			
شُموخ	Pride	/ʃumu: x /	2
ُستاءون	Upset	/mustaʔu:n /	3
يَفْزعونَ	Frightened	/ yafzaʕu:na /	4
Phonemic segmentation			
شَمْس	Sun	/ʃams/	1
جَرَس	Bell	/garas/	2
قَلَم	Pen	/qalam/	2
Phonemic deletion			
رُز بلا - ز	/rice/ without /c /	/ ruz / without /z/	1
مَريض بلا – م	/sick/ without /s /	/mari:d̪ˁ / without /m /	2
مسْطَرَه بلا - ط	/ruler/ without /l /	mas t̪ˁara /without /t̪ˁ/	3
Words and verbs inflection			
غ.ن.ى (هم-ماضي) غنّوا	They sang	/ ɣ.n.a: (hum-in past)/ / ɣannaw /	2
ل.ب.س (هنّ-مضارع) يلبسنَّ	They wearing	/l.b.s (hun- in present)/ / yalbasna /	3
حاسوب (هو) حاسوبهُ	His laptop	/ ħasu:b (huwa)/ / ħa:su:bahu /	3
Morphological fluency task			
ج.ل.س	Sit	/ʒ.l.s /	
ن.ظ.ف	Clean	/n. ðˁ.f /	
ب.ر.د	Cold	/b.r.d /	
